# INTEREST GROUP SESSION—WOMEN’S ISSUES: FROM MENOPAUSE TO MEDICARE: CONTRIBUTIONS FROM COHORT STUDIES OF AGING WOMEN’S HEALTH

**DOI:** 10.1093/geroni/igz038.1286

**Published:** 2019-11-08

**Authors:** Nancy F Woods, Barbara B Cochrane, Barbara B Cochrane

**Affiliations:** 1 University of Washington, Department of Biobehavioral Nursing and Health Informatics, Seattle, Washington, United States; 2 University of Washington School of Nursing, Seattle, Washington, United States

## Abstract

Although some countries have well-established birth cohorts for research on aging, few US projects have followed the evolution of women’s health from midlife through older age, leaving gaps in understanding their health and ignoring a window of time spanning age groups during which women may benefit from health promotion and prevention efforts. Cohort studies of midlife and older women’s health have begun to address this gap, providing opportunities to advance our understanding of both reproductive and healthy aging, including multiple racial/ethnic groups and US regions. Studying existing cohorts of aging women provides investigators with opportunities to: track changes over time, identifying trajectories of health along with aging; clarify antecedent-consequent relationships; identify how historical events may be related to emergent patterns of health; incorporate common data elements that allow comparisons within and across cohorts over time; and introduce new measures/indicators, such as genomic markers. This symposium will provide a foundation for future research using existing databases from studies providing longitudinal health-related measures of women as they age. Opportunities for conducting new analyses on midlife and older women’s health will be described for four large cohort studies: two studies of women exclusively -- the Study of Women’s Health Across the Nation and the Women’s Health Initiative – and two studies with large numbers of women participants – the Baltimore Longitudinal Study of Aging and the Rancho Bernardo Study of Healthy Aging. Unique and common considerations for answering future questions about women’s health from menopause to Medicare in these studies will be discussed.

